# RNA-induced epigenetic silencing inhibits HIV-1 reactivation from latency

**DOI:** 10.1186/s12977-018-0451-0

**Published:** 2018-10-04

**Authors:** Catalina Méndez, Scott Ledger, Kathy Petoumenos, Chantelle Ahlenstiel, Anthony D. Kelleher

**Affiliations:** Department of Immunovirology and Pathogenesis, Level 5, Wallace Wurth Building, The Kirby Institute for Infection and Immunity, UNSW Sydney, Kensington, Sydney, NSW 2052 Australia

**Keywords:** HIV-1, Latency, Reactivation, Latent reservoir, Epigenetic silencing, Transcriptional gene silencing, Transcription

## Abstract

**Background:**

Current antiretroviral therapy is effective in controlling HIV-1 infection. However, cessation of therapy is associated with rapid return of viremia from the viral reservoir. Eradicating the HIV-1 reservoir has proven difficult with the limited success of latency reactivation strategies and reflects the complexity of HIV-1 latency. Consequently, there is a growing need for alternate strategies. Here we explore a “block and lock” approach for enforcing latency to render the provirus unable to restart transcription despite exposure to reactivation stimuli. Reactivation of transcription from latent HIV-1 proviruses can be epigenetically blocked using promoter-targeted shRNAs to prevent productive infection. We aimed to determine if independent and combined expression of shRNAs, PromA and 143, induce a repressive epigenetic profile that is sufficiently stable to protect latently infected cells from HIV-1 reactivation when treated with a range of latency reversing agents (LRAs).

**Results:**

J-Lat 9.2 cells, a model of HIV-1 latency, expressing shRNAs PromA, 143, PromA/143 or controls were treated with LRAs to evaluate protection from HIV-1 reactivation as determined by levels of GFP expression. Cells expressing shRNA PromA, 143, or both, showed robust resistance to viral reactivation by: TNF, SAHA, SAHA/TNF, Bryostatin/TNF, DZNep, and Chaetocin. Given the physiological importance of TNF, HIV-1 reactivation was induced by TNF (5 ng/mL) and ChIP assays were performed to detect changes in expression of epigenetic markers within chromatin in both sorted GFP^−^ and GFP^+^ cell populations, harboring latent or reactivated proviruses, respectively. Ordinary two-way ANOVA analysis used to identify interactions between shRNAs and chromatin marks associated with repressive or active chromatin in the integrated provirus revealed significant changes in the levels of H3K27me3, AGO1 and HDAC1 in the LTR, which correlated with the extent of reduced proviral reactivation. The cell line co-expressing shPromA and sh143 consistently showed the least reactivation and greatest enrichment of chromatin compaction indicators.

**Conclusion:**

The active maintenance of epigenetic silencing by shRNAs acting on the HIV-1 LTR impedes HIV-1 reactivation from latency. Our “block and lock” approach constitutes a novel way of enforcing HIV-1 “super latency” through a closed chromatin architecture that renders the virus resistant to a range of latency reversing agents.

**Electronic supplementary material:**

The online version of this article (10.1186/s12977-018-0451-0) contains supplementary material, which is available to authorized users.

## Background

Human immunodeficiency virus type 1 (HIV-1) latent proviruses are not targeted by current therapeutic strategies. Antiretroviral therapy, when commenced very early in infection has shown significant reduction on the size of the reservoir but limited decay beyond 1 year of therapy [1], and cessation of therapy results in rapid recrudescence of plasma viraemia.

Viral reactivation strategies have emerged as possible approaches to eradicate the latent HIV-1 reservoir. However, the successful reactivation of latent HIV-1 ex vivo and in vivo has proven difficult [2–6]. Therefore, novel strategies that aim to permanently block HIV-1 replication warrant investigation. Importantly, eradication of the latent provirus may not be necessary if spontaneous viral reactivation can be thwarted by mechanisms such as inducing or maintaining viral epigenetic silencing, also known as transcriptional gene silencing (TGS) [7, 8].

We have identified two si/shRNAs, 143 [9] and Prom A [10], as inducers of TGS, able to act individually or combined to efficiently suppress HIV-1 replication. The epigenetic mechanism, involves the induction of altered chromatin architecture associated with the recruitment of Argonaute 1 (AGO1), histone deacetylases (HDACs), histone 3 lysine 9 trimethylation (H3K9me3) and histone 3 lysine 27 trimethylation (H3K27me3). The regions targeted by these si/shRNAs in the HIV-1 5′LTR are separated by ~ 200 nt. The target si/shRNA 143 sequence is located upstream of Nuc-0 in a region rich in transcription factor (TF) binding sites, including AP-1 and COUP-TF [7]. Si/shRNA PromA sequence targets the unique NF-κB tandem binding site in the region between Nuc-0 and Nuc-1 [7, 9, 10]. An illustration of the HIV-1 LTR indicating TFs and si/shRNAs can be found in [9].

HIV-1 reactivation from latency can be triggered by signaling through various cellular pathways that induce nuclear translocation of specific TFs to the HIV-1 promoter [11–13]. We hypothesized that given the strategic location of the si/shRNAs target sites, each si/shRNA may vary in their ability to provide protection from different reactivation stimuli. Consequently, combined expression of both si/shRNAs may provide broader protection against diverse endogenous and exogenous stimuli that threaten to reactivate latent HIV-1. We reasoned that resistance to HIV-1 reactivation might originate from the continuous re-establishment of repressive epigenetic marks induced by the constant supply of each siRNA from an shRNA cassette, even during treatment with LRAs. Therefore, we evaluated the ability of these single shRNAs and their combined expression to impede HIV-1 reactivation from latency, despite treatment with various reactivation stimuli, and characterized the epigenetic profile associated with resistance to reactivation.

## Methods

### shRNAs and lentiviral vectors

ShRNAs were cloned into the lentiviral vectors psi-LVRU6MP or psi-LVRU6MH (GeneCopoeia, Rockville, MD), with an mCherry reporter [9]. ShRNAs: PromA (GGGACTTTCCGCTGGGGACTTCTGTGAAGCCACAGATGGGAAGTCCCCAGCGGAAAGTCCC, targets region 350–370); 143 (GCTAGTACCAGTTGAGCCATTCTGTGAAGCCACAGATGGGAATGGCTCAACTGGTACTAGC, targets region 143–163); locations are based on HXB2 genomic coordinates. Specificity controls included shRNA sequences containing specific mutations in “seed regions” of target sequences of PromA and 143: M2 for PromA (GGGACTTTaaGCTGGGGACTTCTGTGAAGCCACAGATGGGAAGTCCCCACttAAAGTCCC) [14] and 143_3M for 143 (GCTAGatCCgGTTGAGCCATTCTGTGAAGCCACAGATGGGAATGGCTCAACcGGatCTAGC,); mutations are indicated in lower case; and a scrambled control shRNA CtrL (GCTTCGCGCCGTAGTCTTA, purchased from GeneCopoeia). Lentiviral vectors were generated in HEK293 cells using PEI based transduction, as previously described [15]. Herein, the prefix “sh” before each name is used to refer to the shRNA: shPromA, shM2, sh143, sh143_3M and shControl.

### Cell culture

J-Lat 9.2 cell line, a model of HIV-1 latency that expresses GFP upon reactivation of full-length provirus [16], and Jurkat E6 cells were obtained through the NIH AIDS Reagent Program, Division of AIDS, NIAID, NIH: J-Lat 9.2 (Cat. No. 9848) from Dr. Eric Verdin [16] and Jurkat E6-1 (Cat. No. 177) from Dr. Arthur Weiss [17]. Cells were grown in RPMI supplemented with 10% FCS (Gibco^®^, ThermoFisher Scientific, Massachusetts, USA), 8 mM GlutaMax, 5 U/mL penicillin and 50 mg/mL streptomycin (Life Technologies, ThermoFisher Scientific, Massachusetts, USA). Transduced cells were grown under selection with 400 μg/mL of Hygromycin-B (PromA), 1 μg/mL of Puromycin (143, 143_3M, M2 and Ctrl) or both (cells transduced with PromA and 143 from independent lentiviral vectors). For convenience, the name of the stably transduced shRNA is used to refer to the transduced cell line: PromA, 143, PromA/143, M2, 143_3M, Control. PromA/143 is abbreviated to A/143 only in the figures. The terms, Parental and E6, are used to refer to the untransduced J-Lat 9.2 and Jurkat E6 cell lines, respectively.

The DNA sequence of each shRNA was confirmed by sequencing of cellular DNA. Transduced cell lines were purified via sorting based on their mCherry expression after three weeks of lentiviral transduction.

### Quantitation of shRNA expression

RNA was extracted from all JLat 9.2 cell lines using Monarch Total RNA Miniprep kit as per the manufacturer’s instructions (NEB, Cat# 2010S). RNA was quantitated by Nanospectrometer and 1000 ng of RNA was transcribed using the miScript II RT Kit as per the manufacturer’s protocol (Qiagen, Cat# 218161). cDNA was diluted as per kit instructions using nuclease free H_2_O (from 20 μL to 500 μL volume). qPCR was then performed using miScript SYBR Green PCR kit (Qiagen Cat# 218073). Primer pairs used were a universal loop primer 5′-TTC TGT GAA GCC ACA GAT GGG AA-3′ and the Qiagen universal reverse primer supplied with the miScript kit. The qPCR was run on a Roche LightCycler 480 using the following cycle; initial step 95 °C 15 min, 45 cycles of 94 °C for 10 s, 58 °C for 20 s, 70 °C for 20 s. Expression was normalised to the Hs_RNU6-2_11 referencing gene (QIAGEN, #MS00033740).

### Viral Quantitation

Reverse Transcriptase activity (RT-assay) in culture supernatants and Cell-associated HIV-1 *gag*-mRNA were quantitated as described [18, 19]. Integrated HIV-1 was detected using a nested real-time HIV-1 Alu PCR, as described [20]. Detection of latent HIV-1 and HIV-1 reactivated from latency was performed via flow cytometry on Parental and transduced J-Lat 9.2 cell lines (See Flow cytometry section).

### Infection of Jurkat E6 cell lines

A total of 3 × 10^5^ untransduced or transduced Jurkat E6 cells were seeded in 12-well plates and infected with 375 μU/mL RT activity of HIV-1_SF162_. Infection proceeded for a period of 10 days, during which samples were collected for RT-assay. At day 10 cells were harvested for RNA and DNA extraction.

### Drug treatments

In reactivation experiments concentrations of TNF (R&D Systems, Inc. Minneapolis, USA) ranged from 0.001 to 100 ng/mL, in 1:2 dilution series. Bryostatin (Sigma-Aldrich Co. Missouri, USA.) was used at 1, 5 and 10 ng/mL, each in combination with 5 ng/mL of TNF. Suberoylanilide Hydroxamic Acid (SAHA) (Sigma-Aldrich Co. Missouri, USA) was evaluated at concentrations of 0.001 to 100 μM, and when combined with TNF the range was adjusted to a maximum of 25 μM. Chaetocin (Sigma-Aldrich Co. Missouri, USA) was used at 0, 25, 50, 100 nM. DZNep (Sigma-Aldrich Co. Missouri, USA) was used at 0, 25, 50, 100 μM. No-drug controls contained the diluent used to dissolve each drug alone.

### Reactivation experiments and flow cytometry

Treatments were added for 48 or 72 h to 50,000 cells per well in 96-well plates. Following treatment, the wells were washed once with 100 μL of cold DBPS, centrifuged at 500*g* at 4 °C for 1 min and resuspended in 50 μL of DPBS containing 1 μL/mL of LIVE/DEAD^®^ Fixable Near-IR Dead cell stain for 633/635 nm to stain dead cells following manufacturer’s instructions (Thermo Fisher Scientific Inc. (NSYE: TMO)), and fixed in 100 μL of 0.5% PFA. High throughput flow cytometry was performed directly from the 96-well plates using a BD LSRFortessa™ SORP cell analyser using the BD™ High Throughput Sampler Option (HTS)-LSRFortessa microplate adaptor and acquisition was performed using the following detection settings: Near-IR from the Red laser 780/60-A [642 nm], mCherry from the Yellow-Green laser 610/20-A [561 nm] and GFP from the Blue laser 530/30-A [488 nm]. Reactivation from latency was measured only in live single-cells by negative gating of dead cells, followed by gating on mCherry^+^ (transduced cell lines only), and then GFP^+^ or GFP^−^. Reactivation from HIV-1 latency was quantitated as the percentage of GFP positive cells and as the mean fluorescent intensity (MFI) of the GFP signal.

### Cell sorting of mCherry^+^/GFP^+^ and mCherry^+^/GFP^−^ cells

A total of 1 × 10^7^ transduced J-Lat 9.2 mCherry^+^ cells per transduced cell line were resuspended in 20 mL of supplemented RPMI containing 5 ng/mL of TNF, for 48 h. After 48 h cells were washed and stained with LIVE/DEAD^®^ Fixable Near-IR Dead cell stain. The live, Near-IR^−^/mCherry^+^ cells were sorted into GFP^+^ and GFP^−^ populations, and pellets immediately processed using the Magna ChIP™ HT96 Chromatin Immunoprecipitation Kit (Merck-Millipore, Darmstadt, Germany). Cell sorting was performed in a BD Biosciences Influx v7 cell sorter using the color channels 750/LP [640 nm] for Near-IR Live/Dead fixable dye, 610/20 [561 nm] for mCherry and 545/27 [488 nm] for GFP.

### ChIP assays

Chromatin was sheared into fragments of ~ 200 bp using a QSonica 700 sonicator at 4 °C at 50% power, for 15 min (1 min ON, 1½ min OFF), with an internal threshold shutdown temperature of 12 °C. Immunoprecipitations (IP) were performed in duplicates from biological replicates in 96-well plates using 3 μg/mL of antibody with 10 μL of magnetic beads per IP, in a final volume of 100 μL per well, following manufacturer’s instructions. Each IP contained 8 × 10^4^ cell equivalents from sorted mCherry^+^/GFP^+^ HIV-1 reactivated cells or 1 × 10^5^ cell equivalents from mCherry^+^/GFP^−^ HIV-1 latent cells. Each plate included No-Antibody controls per chromatin sample to correct background signal from IPs performed with antibodies of different isotypes and/or specificities.

The following antibodies were used for ChIP assays; Anti-AGO1 clone 4G7-E12 (Cat. No. MABE143), ChIPAb + Acetyl-Histone H3 (Lys9) (Cat. No. 17-658), ChIPAbTM + Trimethyl-Histone H3 (Lys9) (Cat. No. 17-625), ChIPAb + Trimethyl-Histone H3 (Lys27) (Cat No. 17-622), ChIPAbTM + HDAC1 (Cat. No. 17-10199), ChIPAb + TM Trimethyl-Histone H3 (Lys4) (Cat No. 17-614), Anti-RNA polymerase II subunit B1 (phospho CTD Ser-2) Antibody clone 3E10 (Cat No. 04-1571), and Anti-RNA polymerase II subunit B1 (phospho-CTD Ser-5) clone 3E8 (Cat No. 04-1572). All antibodies were purchased from Merck-Millipore (Darmstadt, Germany).

DNA eluted from the HIV-1 LTR region targeted by shPromA/sh143 was quantified by real time PCR as previously described [10]. Percent of Input was used to calculate the amount of immunoprecipitated DNA, and was either used as an absolute, or as a relative value normalized to the Parental cell line.

### Statistical analyses

Data from HIV-1 reactivation assays were analysed using the non-parametric Kruskal–Wallis test corrected for multiple comparisons using the Dunn’s test with adjusted *P* value and results are shown as mean ± SD. Data from the shRNA expression assay was analysed using the non-parametric Wilcoxon matched-pairs signed rank test with results shown as mean ± SD. ChIP data were analysed by performing an Ordinary two-way ANOVA followed by post hoc Holm Šídák multiple comparison tests (See Additional file 1: Statistical analyses, for details). *P* ≤ 0.05 was the minimal threshold for determining statistical significance. The different levels of significance are represented in each plot and explained in the figure legends. Analyses were performed using Prism Version 6.0 (Graphpad Software, San Diego, CA).

In the ANOVA the dependent variable is the relative quantity of DNA that immunoprecipitated with each antibody, measured as the normalised or absolute % of Input. For AGO1, HDAC1 and epigenetic marks analyses, the % Input was normalized to the Parental control. The two independent categorical variables or factors are “cell line” and “condition”. The analysis involves interaction and comparisons between the factors. The factor “cell line” refers to each of the six shRNA-transduced J-Lat 9.2 cell lines plus the corresponding Parental control. The factor “condition” refers to the transcriptional state of the HIV-1 promoter as either in latency or during reactivation treatment with TNF. We performed two types of comparisons using the standard ANOVA terminology: (1) “in-between” cell lines, in which the effect of the shRNA from each cell line is compared to the Parental cell line within the same condition (either pre or post reactivation treatment); and (2) “in-within” cell lines, in which the effect of the shRNA is compared between the two conditions within each cell line in order to compare the epigenetic changes that occur from latency to reactivation (pre vs post treatment with TNF). In contrast, in the studies of bivalency and phosphorylation states of RNA Pol II, data were analysed using an ordinary two-way ANOVA comparing the absolute % Input of two epigenetic or chromatin-associated marks, only during HIV-1 latency. Thus, factor two now corresponds to the epigenetic- or chromatin-associated marks whose absolute levels are being compared: H3K4me3 compared to H3K27me3, and RNA Pol II pSer2 compared to RNA Pol II pSer5.

## Results

### TGS-inducing shRNAs protect Jurkat E6-1 cells from HIV-1_SF162_ replication

**Fig. 1:**
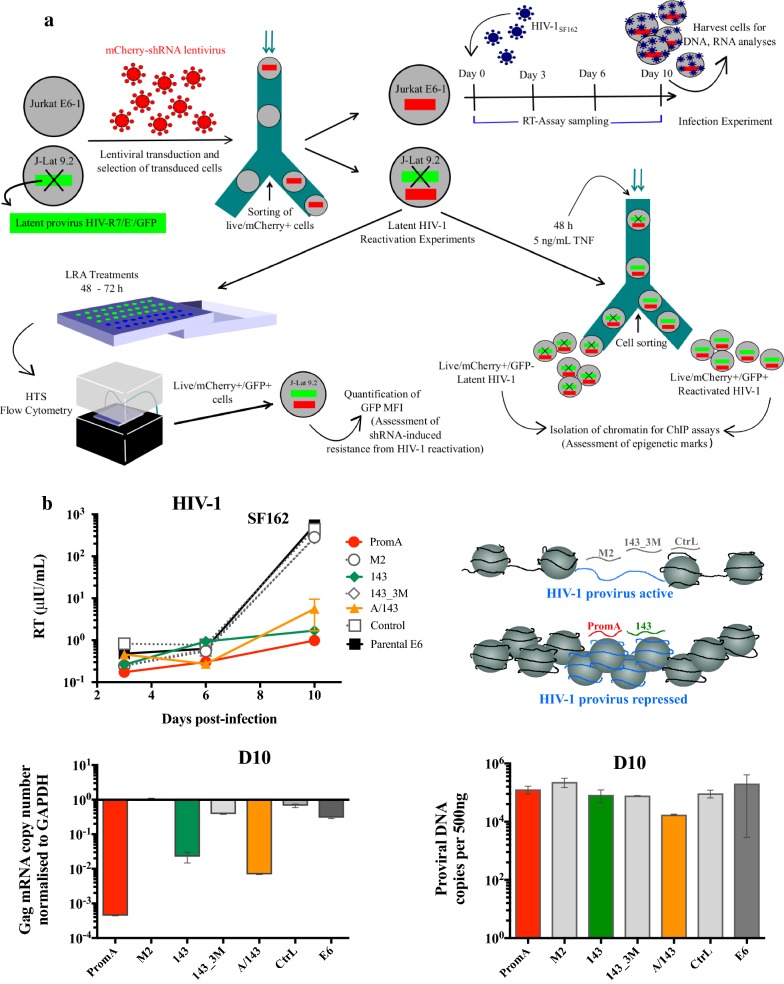
Protective shRNAs inhibit HIV-1_SF162_ replication in Jurkat E6 cells. **a** Diagram illustrating the conduct of experiments. For simplicity, Parental and E6 untransduced cell lines are not indicated, however these cells underwent the same experimental procedures with the exception of the gating strategy for flow cytometry, as these do not express mCherry protein. **b** Upper left panel: Parental and transduced Jurkat E6 cells were infected with 375 μU/mL of HIV-1_SF162_ for 10 days and RT-assays performed in duplicates at the indicated time points post-infection. Lower left panel: Cells were harvested at D10 and cell-associated HIV-1 *gag*-mRNA was quantitated in duplicates via qRT-PCR and were normalised against *GAPDH* mRNA. The dotted line at 10° = 1, indicates the level at which transcription of *GAPDH* and HIV-1 *gag*-mRNA is equivalent. Upper right panel: Schematic of chromatin structure following siRNA treatment. Lower right: Levels of integrated HIV-1 were quantitated at D10 using a HIV-1 nested real-time Alu PCR. Second-round PCRs were performed in triplicates and are presented normalised to β-actin. Data are presented as the number of HIV-1 integrated DNA copies per 500 ng of DNA, with 500 ng equivalent to the DNA amount of 80,000 cells. Data are from one time-course performed with duplicate biological replicates and are shown as Mean ± SD

To confirm the shRNAs silencing activity we performed a time-course of infection on Jurkat E6 cells (Fig. 1a, top right) and measured the levels of HIV-1 replication via RT-assays, qRT-PCR of HIV-1 *gag*-mRNA and qPCR of Alu HIV-1 integrated DNA. Suppression of viral replication by 100% matched shRNAs in this setting, is expected to translate as resistance to reactivation during latency. Previous experiments in our laboratory have demonstrated that stable expression of PromA in MOLT-4 cells provides prolonged and potent protection for over 1 year [21]. Therefore, to further challenge and possibly disrupt RNA-induced epigenetic silencing in cells expressing TGS-inducing shRNAs, we used an extremely high amount of HIV-1_SF162_ virus, 1125 mU/uL per 3 × 10^5^ cells. This also increased the likelihood of having all the cells infected with at least one provirus. Despite the high virus inoculum, on Day 10 (D10) there was at least 10-fold difference in the levels of RT activity between controls and protected cell lines (Fig. 1b, upper left panel), confirming that PromA, 143 and PromA/143 combined expressing cells were able to specifically repress HIV-1 transcription (Fig. 1b, upper right panel). A/143 showed some variation at D10, but the levels remained within the same range (> 10 μU/mL) as those of PromA and 143. Similarly, HIV-1 *gag*-mRNA expression was > 1000 times less in PromA, ~ 80 times less in 143, and > 100 times less in PromA/143, compared to the infected parental cell line (Fig. 1b, lower left panel). Also, the levels of integrated HIV-1 DNA were approximately the same in all the cell lines (Fig. 1b, lower right panel). This was not unexpected because the experimental conditions were directed to infect all the cells with at least one viral particle and the shRNAs do not prevent integration of proviral DNA. These data confirmed previous observations that these constructs are able to suppress HIV-1 replication during a robust infection challenge, while 2–3 mismatches in the target shRNA sequence disabled this protective effect.

### Protective shRNAs provide resistance to HIV-1 reactivation during LRA treatments

**Fig. 2:**
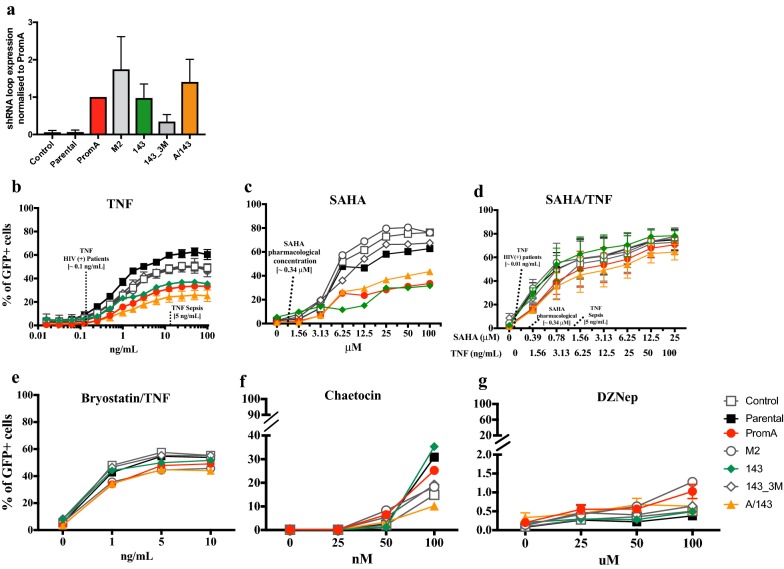
Protective shRNAs inhibit reactivation of latent HIV-1. Parental and transduced J-Lat 9.2 cells were quantified for shRNA expression **a** and then treated with increasing concentrations of **b** TNF, **c** SAHA, **d** SAHA and TNF, **e** Bryostatin and TNF, **f** Chaetocin, **g** DZNep,. All treatments went for 48 h, except from Chaetocin and DZNep, which were for 72 h. Percentage of GFP + cells was measured through flow cytometry from the live (Parental) or live/mCherry + cells (transduced cell lines) and a total of 10,000 events were collected from each sample from the live cell population. The relevant concentrations of TNF and SAHA are indicated in the corresponding plots. Comparisons shown were performed against the Parental cell line using the Kruskal–Wallis Multiple comparison test, correcting for multiple comparison with Dunn’s test (*p ≤ 0.05)(Mean ± SD). Data shown is from at least one independent representative experiment performed in triplicate

We previously showed that stable expression of shPromA, sh143 or shPromA/143 provided protection from HIV-1 reactivation when challenged with TNF, SAHA or a combination thereof [9]. This, in addition to the strong silencing observed during the time course infection, prompted us to challenge the transduced and Parental J-Lat 9.2 cell lines with a panel of potential LRAs (Fig. 2) to assess if combined expression of the two protective shRNAs resulted in stronger or broader protection to a wider range of stimuli.

We first quantitated the levels of shRNA expression in all JLat 9.2 cell lines to determine whether each shRNA construct was expressed to the same degree. We observed similar shRNA expression across all J-Lat 9.2 cell lines, with no significant differences observed (Fig. 2a). The loop sequence was not present in the scrambled Control construct or the untransduced cell line and was therefore not detected in these cell lines.

**Fig. 3:**
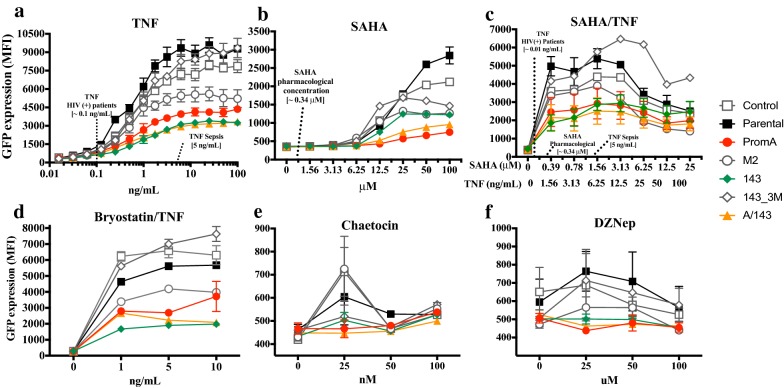
GFP^+^ cells expressing protective shRNAs expressed reduced levels of GFP. Parental and transduced J-Lat 9.2 cells were treated with increasing concentrations of **a** TNF, **b** SAHA, **c** SAHA and TNF, **d** Bryostatin and TNF, **e** Chaetocin, **f** DZNep. All treatments were assessed at 48 h, except from Chaetocin and DZNep, which were assessed at 72 h. Expression of GFP was measured through flow cytometry as the mean fluorescence intensity (MFI) from the population of GFP + cells gated from the live (Parental) or live/mCherry + cells (transduced cell lines), after collecting 10,000 live events. The clinically relevant concentrations of TNF and SAHA are indicated in the corresponding plots. Comparisons shown were performed against the Parental cell line using the Kruskal–Wallis Multiple comparison test, correcting for Multiple comparison with Dunn’s test (*p ≤ 0.05)(Mean ± SD). Data shown is from at least one independent representative experiment performed in triplicates

**Fig. 4:**
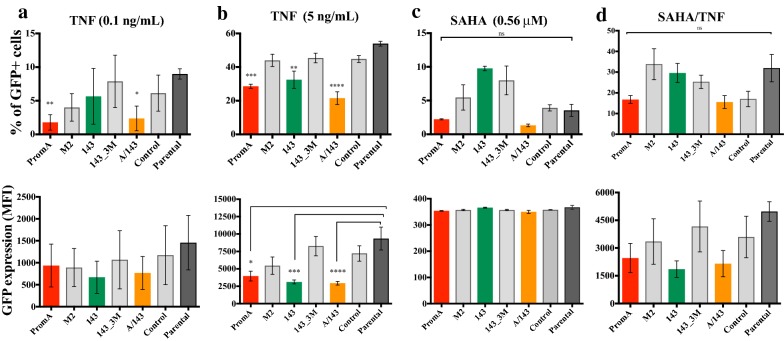
Statistical significance of shRNA-induced protection at concentrations analogous to physiological levels of the LRAs. The levels of GFP expression during HIV-1 reactivation with** a**,** b** TNF,** c** SAHA, or** d** SAHA and TNF at the indicated relevant concentrations, were analysed for a significant decrease in comparison to Parental (dark-grey) by using the non-parametric Kruskal–Wallis Multiple comparison test, correcting for multiple comparisons using the Dunn’s Test with adjusted *P* value. Data shown are from two independent experiments performed in triplicate (Mean ± SD). Significance levels are as follows: * = *p* values from 0.05 to 0.01, ** = *p* values from 0.009 to 0.001, *** = *p* values from 0.0009 to 0.0001 and **** = *p* values < 0.0001

**Fig. 5:**
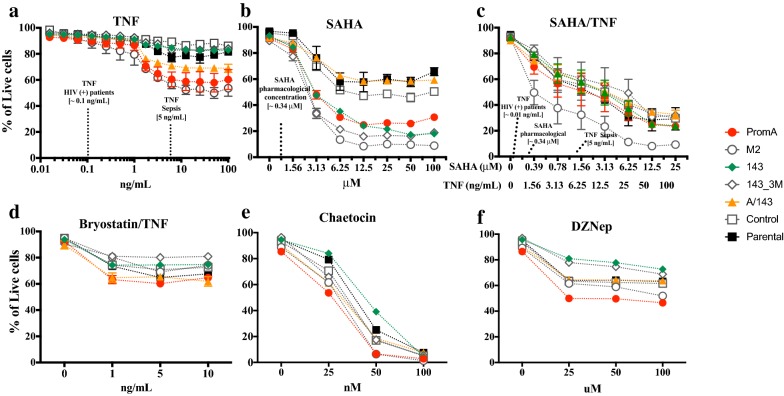
Cell viability during LRA treatment. The percentage of live cells was measured via negative gating of dead cells using the LIVE/DEAD fixable dye. These live cells were further gated to measure the GFP expression levels shown in Figs. 2 and 3, 10,000 live events were collected. **a** TNF, **b** SAHA, **c **SAHA and TNF, **d** Bryostatin and TNF, **e** Chaetocin, **f **DZNep. All treatments were assessed at 48 h, except from Chaetocin and DZNep, which were assessed at 72 h. The clinically relevant concentrations of TNF and SAHA are indicated in the corresponding plots. Comparisons shown were performed against the Parental cell line using the Kruskal–Wallis Multiple comparison test, correcting for Multiple comparison with Dunn’s test (*p ≤ 0.05)(Mean ± SD). Data shown is from at least one independent representative experiment performed in triplicates

After treatment with TNF the protected cells lines showed the lowest levels of GFP^+^ cells (Fig. 2b) and the lowest levels of GFP expression (MFI) (Fig. 3a), both compared to the parental cell line and specificity controls. This indicates decreased proviral reactivation and decreased transcriptional activity from reactivated proviruses. The highest concentration of TNF in viremic HIV-1 infected patients is 100 pg/mL(0.1 ng/mL), (27), while 5 ng/mL is the highest reported during fatal acute sepsis (37, 62). At 0.1 ng/mL the proportion of GFP^+^ cells in PromA and PromA/143 cells was significantly less compared to Parental (Fig. 4a, left panel), though, the overall extent of reactivation was extremely low across all cell lines and therefore not significant (Fig. 4a, lower). At 5 ng/mL of TNF, both the percentage of GFP^+^ cells and levels of GFP expression were significantly lower in PromA, 143 and PromA/143 (*p* = 0.0005 and *p* = 0.02, *p* = 0.003 and *p* = 0.0005, and *p* < 0.0001 both, respectively) (Fig. 4b). In addition, cell viability was slightly more affected in PromA and M2, than any of the other cell line (Fig. 5a).

We then examined the effect of SAHA, a pan-histone deacetylase inhibitor (HDACi) [22, 23] which has been extensively studied in vivo as an HIV-1 LRA [3, 4]. A concentration of ~ 0.335 μM has been previously used as an in vitro equivalent of 400 mg, the protein-unbound pharmacological concentration after single dose of Vorinostat(ZOLINZA®) (63–64), also known as SAHA. At ~ 0.56 μM SAHA, the nearest to 0.335 μM tested, we found no significant differences in the % GFP^+^ cells nor in the levels of GFP expression (Figs. 2c, 3b, 4c). At higher concentrations the protected cell lines showed the lowest proportion of reactivated cells, and these cells had reduced GFP expression, with PromA and A/143 showing ~ 3-fold less compared to Parental (Figs. 2c, 3b). Interestingly, while 143 showed the least proportion of reactivated cells, these cells were expressing GFP at levels that paralleled those of the M2 control cell line, indicating that the sh143 induced-TGS is more susceptible to SAHA reactivation (Figs. 2c, 3b). Overall, SAHA reactivated a greater proportion of cells from the unprotected cell lines compared to TNF and the induced expression was much lower (Figs. 2c, 3b). Additionally, viability was substantially affected at higher concentrations of SAHA (Fig. 5b).

We next combined TNF with SAHA aiming to disrupt silencing via two different reactivation pathways. We decreased the dose-range of SAHA to evaluate the pharmacologically relevant concentration of ~ 0.335 μM in combination with a concentration of TNF that falls within the high range observed in the plasma of HIV-1^+^ patients (1.5 ng/mL) [24]. Increasing concentrations of the combined treatment SAHA/TNF induced the highest percentage of GFP^+^ cells in all the cell lines with A/143 showing the lowest (Figs. 2d, 3c, 4d), and were increasingly toxic (Fig. 5c). Although, not significant, PromA, 143 and PromA/143 showed ~ 2 fold lower GFP expression and thus less proviral reactivation compared to Parental, up to supra physiologic concentrations of 3.13 μM for SAHA and 12.5 ng/mL for TNF (Fig. 3c). After this point, GFP expression declined in all the cell lines.

Activation of PKC pathways in conjunction with NF-kB can induce potent reactivation of latent HIV-1. Therefore, we sought to induce activation of different PKC isoforms along with the NF-κB pathway by treating the cells with increasing concentrations of Bryostatin [25] in combination with a fixed concentration of TNF (5 ng/mL). The combined treatment reactivated comparable percentages of GFP^+^ cells in all the cell lines (Fig. 2e). However, the protected cell lines showed the lowest GFP expression levels revealing impairment of proviral transcription, while the controls revealed increased GFP expression at all concentrations tested (Figs. 2e, 3d). Cell line 143 appeared less susceptible to disruption by the combined treatment than PromA. In addition, cell viability was comparable across all cell lines (Fig. 5d).

In order to obtain insight into the epigenetic profile induced by the protective shRNAs, we investigated the effect of inhibiting the histone lysine methyltransferases (HKMTs), SUV39H1 and EZH2, using Chaetocin [26] and DZNep [27], respectively. These have been previously used to indirectly assess the epigenetic profile of latent HIV-1 [26, 27]. SUV39H1 mediates the trimethylation of lysine 9 in histone 3 (H3K9me3,) whereas EZH2 mediates the trimethylation of lysine 27 in histone 3 (H3K27me3) [28]. Both, Chaetocin and DZNep were not efficient reactivation treatments under the conditions tested. Chaetocin at its highest concentration reactivated a maximum of ~ 40% in the Parental cell line, 143 and PromA, while A/143 showed the least percentage of reactivated cells (Fig. 2f). Expression was only detected in the specificity controls at 25 nM, while the protected cell lines did not show expression at any concentration (Fig. 3e). Chaetocin was highly toxic at its highest concentrations (Fig. 5e). DZNep was the least efficient treatment for reactivation of latent HIV-1, reactivating a maximum of ~ 1% GFP^+^ cells in PromA and M2 at 100 μM (Fig. 2g). GFP expression was low, but only detected for the specificity controls and the Parental cell lines with peak effect at 25 nM, while undetected in PromA, 143 and PromA/143 across the concentrations tested (Fig. 3f). Viability was mostly affected in PromA (Fig. 5f).

Altogether, the shRNAs showed a differential ability to protect the cells from HIV-1 reactivation depending on the stimuli. The data indicated that protective shRNAs inhibit HIV-1 reactivation at concentrations of LRAs considerably beyond those likely to be relevant in vivo, and that dual expression of shPromA and sh143 generally provided broader protection across a variety of stimuli.

### TGS-inducing shRNAs recruit AGO1 and HDAC1, and maintain the epigenetic repressive mark H3K27me3 at the HIV-1 promoter during TNF-induced reactivation

To investigate the epigenetic changes occurring at the HIV-1 promoter during the LRA challenges, we treated all J-Lat 9.2 cell lines for 48 h with 5 ng/mL of TNF; equivalent to the highest pathological TNF concentration reported in human serum during sepsis [29]. We used this higher concentration to induce levels of GFP expression detectable via ChIP assays enabling the comparison of epigenetic marks between reactivation and latency. We performed ChIP assays on sorted live-GFP^+^ or GFP^−^ cells (Fig. 1a, lower right), and analysed changes in the expression of several markers of heterochromatin associated with the 5′LTR using an ordinary two-way ANOVA to compare the normalized % Input to determine whether the shRNAs modified epigenetic profiles. We use the term “latent” when referring to the sorted GFP^−^ population in which the provirus had not been reactivated, and “reactivated” when referring to the GFP^+^ population in which the provirus reactivated from latency following TNF treatment.

Significant interactions between the effects of the cell lines (shRNA-transduced or Parental) and the condition (latent or reactivated) were identified in the epigenetic profile of the HIV-1 LTR, for the relative presence of AGO1, HDAC1, H3K27me3, H3K9me2 and H3K9me3, but not H3K9Ac. Simple main effects analyses indicated significant differences within each of the factors, “in between” cell line and condition, for all these epigenetic related marks and proteins (Additional file 1: Table A1). These data indicate that some shRNAs modify the epigenetic profile of HIV-1 during latency, during TNF reactivation or during both. The specific interactions between cell lines and conditions were further identified (See Additional file 1: Table A2 for the Summary of *P* values) and are explained below.

**Fig. 6:**
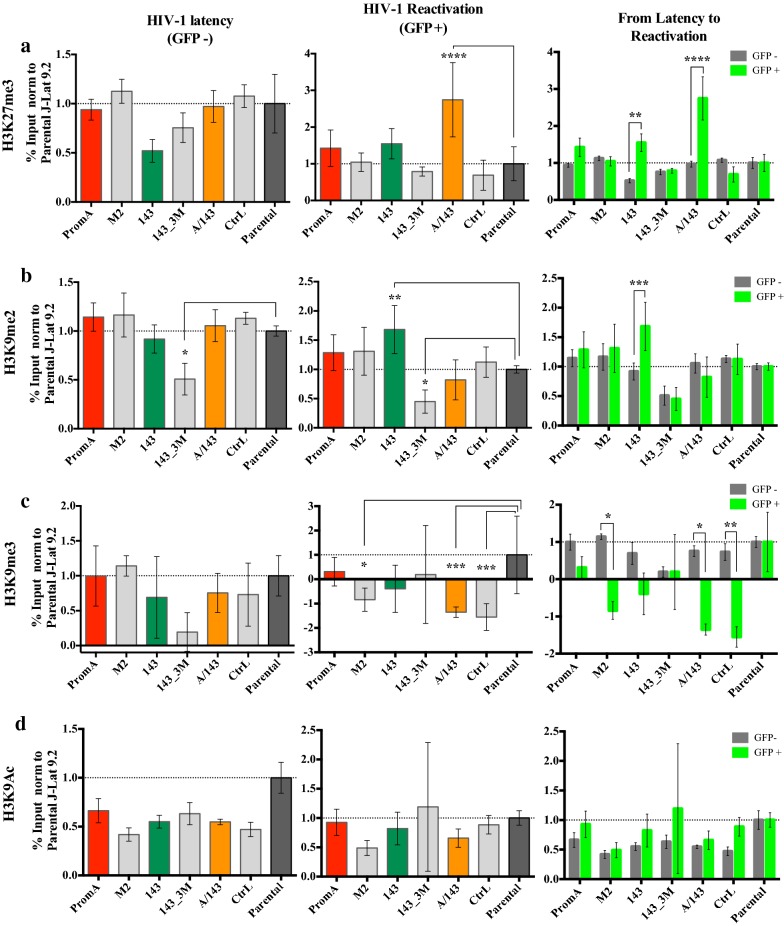
TGS-inducing shRNAs affect the epigenetic profile of the HIV-1 LTR during TNF reactivation. Parental and transduced J-Lat 9.2 cell lines were stimulated for 48 h with 5 ng/mL of TNF, and ChIP assays performed on sorted live GFP- and GFP + populations. The statistical significance of immunoprecipitation levels of **a** H3K27me3, **b** H3K9me2, **c** H3K9me3, and **d** H3K9Ac, during HIV-1 latency (left panels) and HIV-1 reactivation (middle panels), was analyzed by performing “in-between” cell lines multiple comparisons against Parental (dark-grey bar). Multiple comparisons “in-within” cell lines were also performed to examine significant changes from latency to reactivation within each cell line (right panels). Data shows Mean ± SD (n = 4) from two independent ChIP assays and are presented as the % Input normalised to Parental (* = *p* values from 0.05 to 0.01, ** = *p* values from 0.009 to 0.001, *** = *p* values from 0.0009 to 0.0001 and **** = *p* values < 0.0001)

We first evaluated the changes in the levels of repressive and activating epigenetic marks. “In between” comparisons did not find significant differences in levels of H3K27me3 between any of the transduced cell lines and Parental, during latency (Fig. 6a, left). In contrast, PromA/143 demonstrated significantly higher levels of H3K27me3 during TNF driven HIV-1 reactivation when compared to Parental (Fig. 6a, middle) (*p* < 0.0001) indicative of an overdrive mechanism maintaining closed chromatin despite the reactivation stimulus. This provides an explanation of how reactivation is limited in these circumstances. In addition, H3K27me3 levels were considerably higher in PromA/143 during reactivation compared to the levels in latency (*p* < 0.0001) (Fig. 6a, right). In contrast 143 did not show any difference in H3K27me3 levels during reactivation by TNF compared to the parental cell line (Fig. 6a, left and middle), but did show a significant increase of this mark when comparing between latency and TNF reactivation (*p* = 0.004)(Fig. 6a, right). The levels of H3K27me3 in PromA were not different to Parental during both conditions (Fig. 6a, left and middle), nor post activation compared to latency (Fig. 6a, right).

Only 143_3M had significant lower levels of H3K9me2 in comparison to Parental during HIV-1 latency (*p* = 0.04) (Fig. 6b, left) and showed a significant decrease during HIV-1 reactivation (*p* = 0.01) (Fig. 6b, middle). Intriguingly, H3K9me2 was not affected by TNF treatment in any of the other cell lines, except in the 143, in which it showed an increase compared to Parental (*p* = 0.002) (Fig. 6b, right).

We found no differences in the levels of H3K9me3, across cell lines during HIV-1 latency when compared to Parental (Fig. 6c, left). However, upon TNF stimulation M2, PromA/143 and Control cell lines completely lost this epigenetic mark from the HIV-1 promoter (Fig. 6c, middle). In fact, the levels of this mark were so low for these cell lines that the calculated relative % Input fell below the background and hence the negative values indicate a profound depletion of the epigenetic mark. When comparing the levels of H3K9me3 between HIV-1 latency and HIV-1 reactivation in each cell line, all the transduced cell lines appeared to show a decrease upon treatment with TNF, though this decrease was only significant in M2, PromA/143 and Control (Fig. 6c, right).

Consistent with the more limited interaction of H3K9me3 between the two factors, cell lines and transcriptional conditions, there were no significant differences in the levels of H3K9Ac in any of the conditions, or between latency to reactivation (Fig. 6d, Additional file 1: Table A2). This indicates that acetylation of this residue is not affected by the addition of the shRNAs during latency and that TNF treatment has the same effect on all the cell lines tested.

**Fig. 7:**
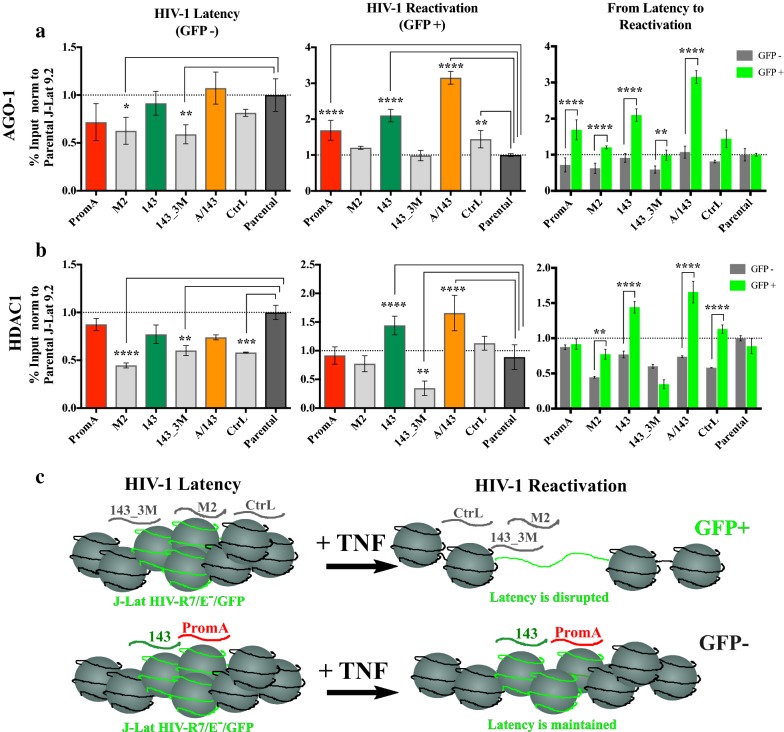
Protective shRNAs recruit AGO1 and HDAC1 during HIV-1 reactivation with TNF. Parental and transduced J-Lat 9.2 cell lines were stimulated for 48 h with 5 ng/mL of TNF, and ChIP assays performed on sorted live GFP- and GFP + populations. Significance of immunoprecipitation levels of **a** AGO1 and **b** HDAC1 during HIV-1 latency (left panels) and HIV-1 reactivation (middle panels), was analysed by performing “in-between” cell lines multiple comparisons against Parental (dark-grey bar). Multiple comparisons “in- within” cell lines where also performed to examine significant changes from latency to reactivation for each cell line (right panels). Data are presented as the % Input normalised to Parental from two independent ChIP assays (Mean ± SD)(n = 4)(* = *p* values from 0.05 to 0.01, ** = *p* values from 0.009 to 0.001, *** = *p* values from 0.0009 to 0.0001 and **** = *p* values < 0.0001). **c** Upper panel: Schematic of latency disruption during HIV-1 reactivation with TNF. Lower panel: Schematic of latency maintenance by TGS-inducing si/shRNAs during treatment with TNF. ShA/143 is not explicitly illustrated because PromA/143 cell line simultaneously co-expresses each, PromA and 143, from independent lentiviral cassettes

“In-between” multiple comparisons revealed significantly lower levels of AGO1 at the HIV-1 LTR for the specificity controls M2 (*p* = 0.01) and 143_3M (*p* = 0.005) cell lines during HIV-1 latency, when compared to Parental (Fig. 7a, left panel). These differences were not observed for PromA, 143 or PromA/143 (Fig. 7a, left panel). In contrast during reactivation, PromA, 143 and PromA/143 showed significant higher levels of AGO1 at the HIV-1 promoter (all *p* < 0.0001), when compared to Parental (Fig. 7a, middle). “In-within” multiple comparisons determined that this increase in AGO1 during reactivation was highly significant (*p* < 0.0001) compared to levels of AGO1 during latency (Fig. 7a, right).

For HDAC1, M2, 143_3M and Control, all showed significant lower levels during latency (*p* < 0.0001, *p* = 0.001 and *p* = 0.0006, respectively), conversely, PromA, 143 and PromA/143, did not (Fig. 7b, left). HDAC1 levels of Control and M2 showed a significant increase from latency to reactivation (Fig. 7b, right) but these levels were no different to those of Parental during TNF reactivation (Fig. 7b, middle); whereas 143 and PromA/143 showed a significant increase during reactivation (both, *p* < 0.0001) (Fig. 7b, middle), and the magnitude of this increase was significant when compared to the levels during HIV-1 latency (Fig. 7b, right).

Together these data support specific recruitment of AGO1 and HDAC1, in addition to H3K27me3, to the HIV-1 LTR by shPromA, sh143 and shPromA/143 during TNF reactivation conditions and is consistent with these constructs acting specifically at the HIV-1 LTR, to maintain epigenetic repression. The protective constructs appear to induce the relative maintenance of certain repressive epigenetic marks despite the presence of drivers of reactivation (Fig. 7c). These results explain how proviral transcription was impaired in the small percentage of protected cells in which the provirus reactivated.

### Maintenance of H3K27me3 is induced by protective shRNAs during reactivation with TNF

**Fig. 8:**
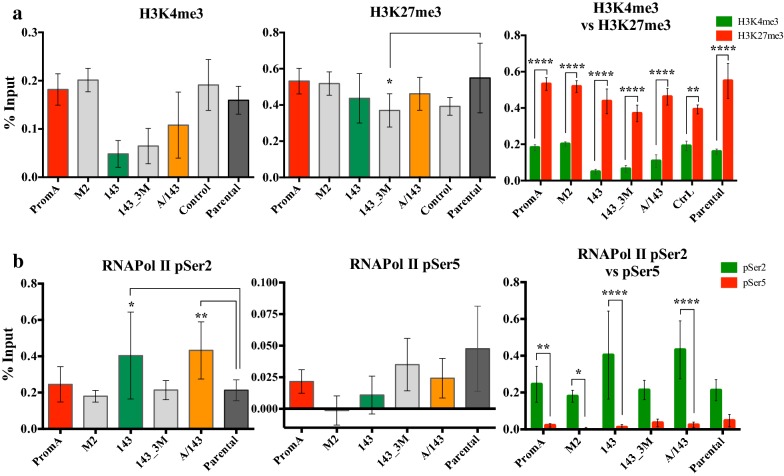
Impact of shRNAs on maintenance of HeK27me3 and RNA Pol II Phosphorylation, during HIV-1 latency. “In-between” cell lines multiple comparisons were used to determine significant differences between the % Input of **a** H3K4me3 (left panel) and H3K27me3 (middle panel), or **b** Phosphorylation of RNA Pol II at pSer2 (left panel) and pSer5 (middle panel), against the levels of Parental (dark-grey). Right panels correspond to “in-within” multiple comparisons between activation (H3K4me3, green) or repression (H3K27me3, red), top-panel; or between elongation (pSer2, green) or stalling (pSer5, red) of RNA Pol II, bottom panel. Data show Mean ± SD (n = 4) from 2 independent ChIP assays. Significance levels are as follows: * = *p* values from 0.05 to 0.01, ** = *p* values from 0.009 to 0.001, *** = *p* values from 0.0009 to 0.0001 and **** = *p* values < 0.0001

The coexistence of H3K4me3 and H3K27me3 in promoter regions is associated with poised or inducible genes (Reviewed in (30)). Their coexistence or bivalency in the HIV-1 promoter may be characteristic of inducible latent proviruses. The ordinary two-way ANOVA did not identify a significant interaction between the cell lines (shRNAs) and these epigenetic marks during latency (Additional file 1: Table A3.). Further, there were no differences in the absolute % Input of H3K4me3 between the transduced cell lines and Parental (Fig. 8a, left and middle). Only 143_3M cell line showed slightly less H3K27me3 compared to Parental cell line (Fig. 8a, middle) (*p* = 0.02).

However, post hoc Holm Šídák multiple comparisons (Additional file 1: Table A4) found significant higher levels of H3K27me3 compared to H3K4me3 within all the cell lines, during HIV-1 latency (PromA, M2, 143 and A/143: p > 0.0001; Control *p* = 0.009) (Fig. 8a, right). These data are consistent with the latent state of the provirus in J-Lat 9.2 cells, suggesting H3K27me3 is imposing a strong repressive signal while H3K4me3 allows for transcriptional activation upon stimulation.

### Phosphorylation of RNA Pol II at Serine 2 of indicates the presence of an elongating polymerase a the Latent HIV-1 promoter

RNA Pol II can be processive or non-processive depending on the phosphorylation state of, Serine 5 (pSer5) and 2 (pSer2) within the Carboxyl-terminal domain (CTD). The phosphorylation status of these residues is differentially associated with initiation and productive elongation of transcription [30, 31]. Statistical analyses identified an interaction between the cell lines and the phosphorylated species of RNA Pol II (*p* = 0.02) (Additional file 1: Table A3). The “in-within” cell line multiple comparisons determined a significant higher % Input of RNA Pol II pSer2, compared to that of pSer5, during HIV-1 latency in PromA (*p* = 0.009), 143 (*p* < 0.0001), PromA/143 (*p* < 0.0001) and M2 (*p* = 0.05) (Fig. 8b, right and Additional file 1: Table A4 top). M2 showed the lowest % Input for both marks (Fig. 8b, left and middle). *Post*-*Hoc* Holm Šídák “in-between” cell line comparisons (Additional file 1: Table A4, bottom) determined the levels of RNA Pol II pSer2 were significantly higher in 143 (*p* = 0.03) and in PromA/143 (*p* = 0.009), compared to Parental (Fig. 8b, left). No significant differences were identified for RNA Pol II pSer5 (Fig. 8b, middle). These data suggest the TGS mechanism induced by shRNAs PromA, 143 and A/143 possibly involves stalling of the elongating pSer2 RNA Pol II early after transcription initiation, interfering with efficient transcription elongation during reactivation stimuli.

## Discussion

Despite effective antiretroviral therapy, HIV-1 provirus persists in the latent reservoir. We have proposed stabilizing HIV-1 latency via a “block and lock” strategy, namely shRNA-induced TGS, as an alternative mechanism of controlling the reservoir. Using the J-Lat model of HIV-1 latency, we stably transfected J-Lats with constructs expressing shRNAs to induce TGS or specificity controls, challenged the induced epigenetic silencing with LRAs possessing different modes of action and assessed the resulting epigenetic profile. Here we have presented evidence that TGS-inducing shRNAs (PromA, 143 and PromA/143) provide robust resistance to reactivation by LRAs in J-Lat 9.2 cells and the impaired proviral gene expression was due to repressive epigenetic mechanisms.

Overall, the protective constructs impeded the reactivation of HIV-1 provirus from a larger population of cells compared to the reactivation events observed in the unprotected cell lines (Fig. 2). Mostly important is that the proviral transcription from the small population of cells expressing the protective constructs was in all cases below the levels measured in the control cell lines (Fig. 3).

Generally, PromA and 143 cell lines appeared more susceptible to HIV-1 reactivation by agents that activated TFs whose binding sites are adjacent to their specific targets in the HIV-1 promoter. Considering that PromA targets the NF-κB binding motifs, some interaction and susceptibility to TNF was expected. Similarly, as sh143 does not target the NF-κB binding motifs, it is not surprising that better protection was observed to TNF than that induced by shPromA, and when combined with shPromA, protection was enhanced. The effect of TNF on HIV-1 promoter during RNA-directed epigenetic silencing is modelled in Fig. 7c.

Interestingly, we observed the opposite during treatment with SAHA, where the 143-transduced cell line showed less protection than PromA. This can be explained in part by the sh143 target site mapping to a cluster of TF binding motifs, specifically AP-1/COUP-TF and NFAT [7, 9], which can provide an anchor for HDAC recruitment [7]. The combination of SAHA/TNF was able to reactivate the provirus in a larger number of protected cells (GFP^+^ cells), consistent with the treatment affecting the target sites of PromA and 143 (Fig. 2d). However, proviral transcription remained impaired demonstrating some level of protection (GFP MFI) (Fig. 3c).

Bryostatin-1 has been tested as an LRA for HIV-1 eradication [6, 25]. It activates not only PKC-α and –δ, through which it is thought to induce reactivation of latent HIV-1 [32], but also induces PKC- ε [33], which promotes T cell survival [34, 35]. In J-Lat 9.2 cells Bryostatin-1 has induced less proviral reactivation than TNF alone [36]. Given that Bryostatin-1 and TNF act via different signaling pathways, we expected the combination of both to be a stronger challenge for our TGS-inducing constructs. Consistent with this, Bryostatin/TNF treatment reactivated comparable number of proviruses in all the cell lines (Fig. 2e), although compared to TNF alone GFP expression was not as high (Fig. 3c). Thus, there seems to be complementarity between these two activators and its combination is able to target more proviruses. Importantly, the protected cell lines showed reduced GFP expression compared to controls indicating the protective shRNAs were impairing proviral transcription (Fig. 3c). Bryostatin inhibits CDK2 through dephosphorylation of threonine 160 [37], indirectly affecting transactivation and interfering with Tat function [38, 39]. Hence, while Bryostatin-1 potentially induces reactivation of latent HIV-1 via activation of PKCs, it simultaneously impairs the HIV-1 transactivation loop by Tat, potentially explaining why the levels of GFP expression were below those of TNF alone.

**Fig. 9:**
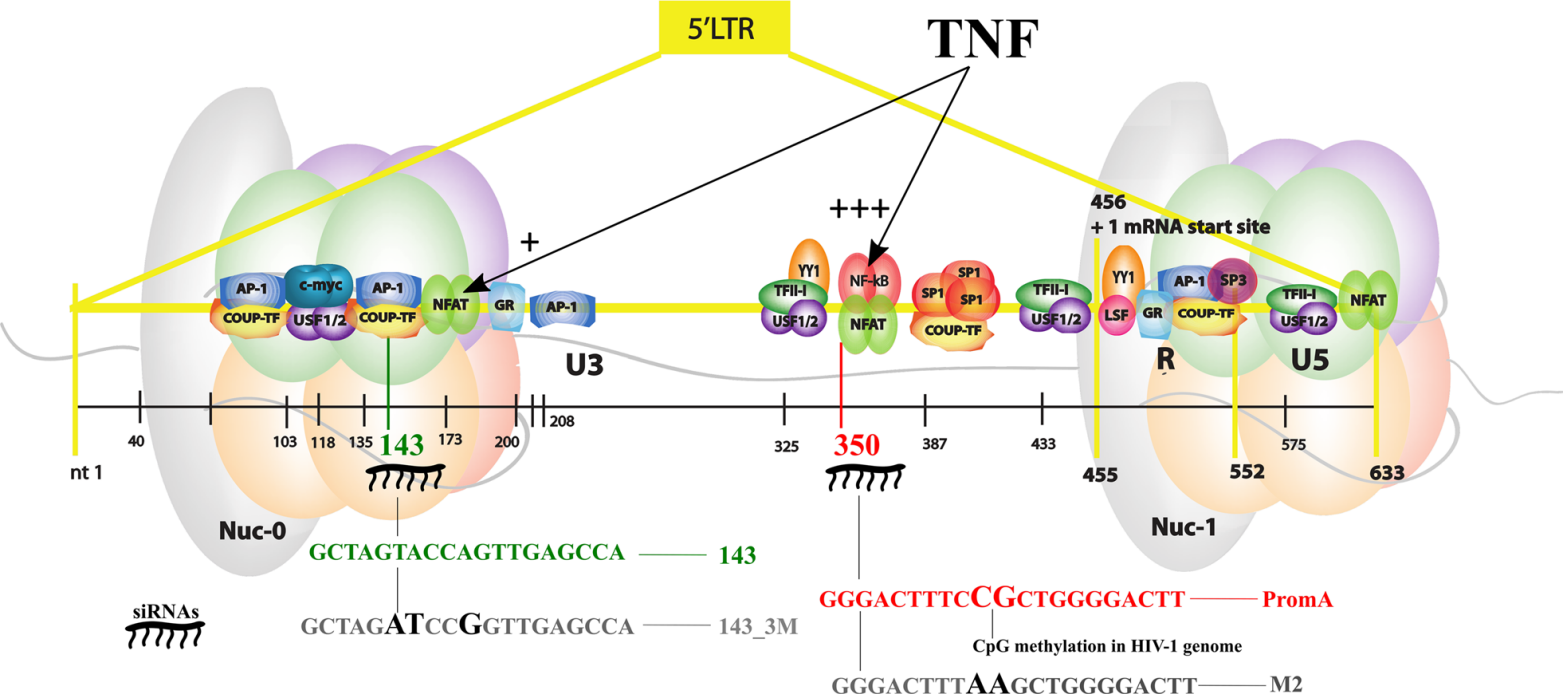
Model of HIV-1 promoter control through RNA-induced epigenetic silencing and the effect of TNF. The two nucleosomes, nuc-0 and nuc-1, are comprised of a histone 1 linker (gray) and four histone pairs (shown in green, orange, purple, and pink) that generate the histone octamer. Activator protein 1 (AP-1), COUP-TF, upstream stimulatory factor 1/2 (USF 1/2), nuclear factor of activated T cells (NFAT), glucocorticoid receptor (GR), transcription factor II-I (TF II-I), Yin Yang 1 (YY1), NF-κB, specificity protein 1 (SP1), late SV40 factor (LSF), specificity protein 3 (SP3)

Thus, PromA and 143 appear to maintain silencing despite reactivation stimuli, but may rely on subtly different epigenetic mechanisms. When both shRNAs are expressed in combination as in PromA/143, they either compensate each other or provide a more stable epigenetic landscape resulting in a more effective lock down of the HIV-1 LTR (Fig. 9).

Treatment with inhibitors of SUV39H1 and EZH2 resulted minimal viral reactivation in the controls at the conditions tested (Fig. 2f,g). Chaetocin seemed to disrupt PromA, 143 and Parental, although not A/143, but expression was barely detected indicating limited contribution of H3K9me3 in the induced-TGS in this setting (Fig. 3e). DZNep resulted in even fewer GFP^+^ cells, but expression was evident in the controls consistent with partial involvement H3K27me3 in maintenance of proviral latency in J-Lat 9.2 (Fig. 3f). Both treatments, in particular Chaetocin, were toxic (Fig. 5e,f), which may be related to the global effect of these ubiquitous methylases in the epigenome.

During LRA treatments, none of the specificity controls were able to provide protection from HIV-1 reactivation, confirming the highly sequence specific nature of shRNA-induced TGS. In stark contrast, the dual construct (shPromA/sh143) broadened the protective effect of the single constructs, demonstrating robust protection from HIV-1 reactivation by modulators of multiple cellular and epigenetic pathways. It was therefore important to examine the epigenetic profiles that occur at the HIV-1 LTR during reactivation from transcriptional control, in the few cells in which it was disrupted. We chose TNF reactivation for these experiments as this is arguably the most powerful and physiologically relevant of activation stimuli tested. We treated the cell lines with 5 ng/mL of TNF because this concentration corresponds to the highest concentration reported in humans during acute sepsis [29], and is sufficiently high to increase the odds of detecting epigenetic changes by ChIP, during low-level proviral reactivation in protected cell lines based on the reactivation data.

The protection from TNF reactivation observed with PromA, 143 and PromA/143 was associated with the recruitment of AGO1 to the HIV-1 promoter. Although PromA had a trend towards higher levels of H3K27me3, H3K9me3 and HDAC1, these increases were not significant compared to Parental (Fig. 2b), consistent with PromA being more susceptible to TNF reactivation than 143 and PromA/143. However, given that PromA still impaired HIV-1 transcription (Fig. 3a), it also indicates that PromA may induce and maintain other epigenetic marks in addition to the previously reported increases in H3K9me2, HDAC1 [10], and H3K27me3 [9, 10].

In contrast, 143 and PromA/143 both showed a remarkable recruitment of AGO1 and HDAC1 during HIV-1 reactivation. PromA/143 had the highest retention of H3K27me3 with no variation in the levels of H3K9me2, whereas the inverse was true for 143 (Fig. 6a,b). Therefore, shPromA and sh143 induced distinct epigenetic profiles and when expressed together, the resulting epigenetic profile is further modified. Complementary to these data, M2 and 143_3M showed reduced levels of AGO1 and HDAC1 during latency compared to Parental, indicating the mismatch sequences may be disrupting latency. In fact, 143_3M also showed decreased levels of H3K9me2 during latency, and loss of HDAC1 recruitment after TNF stimulation, as if inducing transcriptional gene activation (TGA) rather than TGS. Further investigations may shed light on the molecular underpinning of TGA by sh/siRNAs.

The observed increase in AGO1 could be the result of TGS reinforcement compensating for TGS disruption. That is, the levels of AGO1-shRNA complexes required to re-establish TGS during exposure to TNF are higher than those required for maintenance of TGS during cell homeostasis. The increased recruitment may be due to TNF also affecting transcription of other genes, including the lentiviral vector and perhaps indirectly *AGO1*, since AGO1 interacts with RNA Pol II and binds to active promoters [40, 41]. In addition, novel transcriptional [42] and microRNA [43] targets were described for NF-κB in HeLa cells treated with TNF, pending to be fully characterized. Interestingly, most genome-wide binding by NF-κB occurs via non-canonical κB sites [44]. Hence, the possibility exists for an indirect effect on AGO1 within this regulatory network.

Quite surprisingly, H3K9Ac levels did not change. This residue does not seem to be considerably involved in the reactivation of latent HIV-1 from J-Lat 9.2 cells (Fig. 6d), though it may function as an anchor for recruitment of other epigenetic marks that affect transcription activation or elongation. In such a scenario other residues, such as H3K27Ac, may become preferentially acetylated during HIV-1 reactivation from latency. Indeed, the residues H3K4me3, H3K9Ac and H3K14Ac co-exist in promoters of paused genes that employ RNA Pol II and are stalled at initiation of transcription [45]. This pattern is found in developmental genes that require a signal to trigger transcription elongation. Further work is required to see if such patterns exist in the HIV-1 promoter in any of its states of latency.

Like H3K9Ac, the levels of H3K9me3 at the HIV-1 promoter were not different across the cell lines while latent. Only PromA showed apparent retention of this mark upon TNF treatment, while complete loss of this mark was observed in other cell lines. Values of H3K9me3 near background levels were consistent with minor reactivation observed with Chaetocin (Fig. 2e). Thus, it appears this epigenetic mark does not explain the resistance to reactivation observed in protected cell lines.

As expected, we found H3K27me3 and H3K4me3 present within the HIV-1 promoter, indicating bivalency, consistent with a latent, but inducible state of the provirus in the J-Lat 9.2 cells. Importantly, different species of phosphorylated RNA Pol II are associated with coexistence of these two epigenetics marks. For instance, paused RNA Pol II, phosphorylated at Ser5 (pSer5), is present in poised developmental genes that remain repressed as a result of PRC2 [46], and are enriched for H3K4me3 and H3K27me3 [31]. The presence of pSer5 state of RNA Pol II is associated with genes susceptible to rapid reactivation. In contrast, phosphorylation at Serine 2 (pSer2) indicates an elongating polymerase. Therefore, we investigated the levels of different forms of phosphorylated RNA Pol II at the LTR region. We expected higher levels of pSer5 compared to pSer2 for PromA, 143 and PromA/143. However, this was not the case. Intriguingly, 143 and PromA/143 showed significantly higher levels of RNA Pol II pSer2 compared to Parental. Levels of pSer5 are known to decrease towards the 3′ end of active genes while those of pSer2 increase [30]. The higher ratio of pSer2 over pSer5 in all the cell lines suggests that the latent provirus in J-Lat 9.2 cells had already initiated transcription before becoming epigenetically silent through endogenous mechanisms. Stalling of RNA Pol II has been described in highly transcribed genes indicating that transcriptional control can occur at any instance of the process. Therefore, our data implicates a more complex mechanism, in which the shRNAs may be interacting with the transcription machinery along with other chromatin related factors to induce inhibition of transcription during the elongation process. Indeed, RNA Pol III transcribed sncRNAs have been shown to inhibit transcription of RNA Pol II transcribed genes [47, 48]. Thus our shRNAs, which are transcribed by the RNA Pol III, could potentially be directing a similar mechanism, perhaps even acting at the 3′end of the antisense HIV-1 specific long non-coding RNA [49, 50]. Importantly, the detection of epigenetic marks in the LTR may correspond partially or completely to the 3′LTR, as it is not possible to distinguish the 3′ from the 5′LTR in the ChIP assay, based on DNA fragment size and on the positioning of the nucleosomes in this region. It is therefore possible that the protective shRNAs are targeting one or both LTRs. Naturally, this means they can also target the 3′UTR of viral transcripts. However, we have previously reported limited contribution of PTGS via mRNA cleavage [9, 10], hence only translational repression on the 3′UTR of viral transcripts is possible during the time it takes for TGS to be established. PTGS is unlikely to be the main mechanism as HIV-1 rapidly develops resistance and multiplex approaches combining siRNAs targeting viral and cellular sequences are essential to control viral replication via this RNAi pathway (Reviewed in [51]).

For the ChIP assays it was important that latent HIV-1 provirus was present in most, preferably all, of the cell population, given that uninfected cells contribute to background noise. J-Lat 9.2 cells provided a practical way of measuring the epigenetics of virus reactivation and due to their clonal characteristic provided consistency in downstream laboratory techniques. Indeed, it would be interesting to investigate the epigenetic profiles of inducible latent HIV-1 proviruses directly from samples of suppressed HIV-1^+^ patients, however this is technically very challenging. We chose J-Lat 9.2 cells because HIV-1 latency arose spontaneously following infection of Jurkat cells with a full-length virus expressing GFP [16]. Hence, this model resembles one of the mechanisms by which latency may be naturally established in vivo, when latency is epigenetically induced in infected cells that are actively dividing rather than as a result of a transition to quiescence.

The data presented suggest that although the silencing action of two protective constructs is associated with epigenetic changes in the proviral promoter, these changes are qualitatively different indicating subtle variations in the exact underpinning mechanism of each si/shRNA. Given the broad and sustained resistance to HIV-1 reactivation, and the epigenetic profile of PromA/143, this combination shows promise as a potential lead candidate to prevent HIV-1 reactivation. The natural epigenetic profile of latent HIV-1 could be super-enforced by the action of TGS-inducing si/shRNAs and consequently become resistant to reactivation. In this way, the si/shRNAs will not only reproduce a latency-like state in actively replicating HIV-1, but will also thwart reactivation from latency, making HIV-1 less sensitive to naturally occurring reactivation stimuli.

Several studies have used CRISPR-Cas9 technology to target HIV-1 (Reviewed in [52]), including targeting cellular receptors required for HIV-1 entry [53], excising the HIV-1 genome from infected cells [54], and reactivating [55] or eradicating latent HIV-1 [56]. While promising, this approach has limitations, such as efficient targeted delivery to the required cells, choice of vector (viral or non-viral origin), adverse effects arising from the choice of vector and immune reactions, most of which are shared amongst gene therapy strategies. Additionally, resistant variants have emerged as a result of the cellular non-homologous end joining repair pathway (NHEJ) and possibly viral transcription, indicating that continuous expression of a combination of highly conserved gRNAs and Cas9 will be necessary [57].

Additionally, studies have investigated the “block and lock” approach using didehydro-cortistatin A (dCA), a Tat-mediated HIV-1 inhibitor that also induces epigenetic modifications [58].

However, this potential anti-HIV-1 therapy needs to develop a sustained response, as current data shows suppression for ~ 3 weeks post-treatment cessation. The “block and lock” cure strategy presented here has the advantages that HIV-1 is unlikely to develop escape mutations and si/shRNAs will be resistant to reactivation.

## Additional file

**Additional file 1.** Statistical results of the ordinary two-way ANOVA analyses for all epigenetic marks.

## References

[1] Koelsch KK (2011). Impact of treatment with raltegravir during primary or chronic HIV infection on RNA decay characteristics and the HIV viral reservoir. AIDS.

[2] Ho YC (2013). Replication-competent noninduced proviruses in the latent reservoir increase barrier to HIV-1 cure. Cell.

[3] Cillo AR (2014). Quantification of HIV-1 latency reversal in resting CD4 + T cells from patients on suppressive antiretroviral therapy. Proc Natl Acad Sci USA.

[4] Elliott JH (2014). Activation of HIV transcription with short-course vorinostat in HIV-infected patients on suppressive antiretroviral therapy. PLoS Pathog.

[5] Bullen CK (2014). New ex vivo approaches distinguish effective and ineffective single agents for reversing HIV-1 latency in vivo. Nat Med.

[6] Darcis G (2015). An in-depth comparison of latency-reversing agent combinations in various in vitro and ex vivo HIV-1 latency models identified bryostatin-1 + JQ1 and ingenol-B + JQ1 to potently reactivate viral gene expression. PLoS Pathog.

[7] Mendez C, Ahlenstiel CL, Kelleher AD (2015). Post-transcriptional gene silencing, transcriptional gene silencing and human immunodeficiency virus. World J Virol.

[8] Weinberg MS, Morris KV (2016). Transcriptional gene silencing in humans. Nucl Acids Res.

[9] Ahlenstiel C (2015). Novel RNA duplex locks HIV-1 in a latent state via chromatin-mediated transcriptional silencing. Mol Ther Nucleic Acids.

[10] Suzuki K (2008). Closed chromatin architecture is induced by an RNA duplex targeting the HIV-1 promoter region. J Biol Chem.

[11] Nabel G, Baltimore D (1987). An inducible transcription factor activates expression of human immunodeficiency virus in T cells. Nature.

[12] Rohr O (1997). COUP-TF and Sp1 interact and cooperate in the transcriptional activation of the human immunodeficiency virus type 1 long terminal repeat in human microglial cells. J Biol Chem.

[13] Yang X, Chen Y, Gabuzda D (1999). ERK MAP kinase links cytokine signals to activation of latent HIV-1 infection by stimulating a cooperative interaction of AP-1 and NF-kappaB. J Biol Chem.

[14] Suzuki K (2011). Transcriptional gene silencing of HIV-1 through promoter targeted RNA is highly specific. RNA Biol.

[15] Aggarwal A (2012). Mobilization of HIV spread by diaphanous 2 dependent filopodia in infected dendritic cells. PLoS Pathog.

[16] Jordan A, Bisgrove D, Verdin E (2003). HIV reproducibly establishes a latent infection after acute infection of T cells in vitro. EMBO J.

[17] Weiss A, Wiskocil RL, Stobo JD (1984). The role of T3 surface molecules in the activation of human T cells: a two-stimulus requirement for IL 2 production reflects events occurring at a pre-translational level. J Immunol.

[18] Suzuki K (1993). Poly A-linked colorimetric microtiter plate assay for HIV reverse transcriptase. J Virol Methods.

[19] Suzuki K (2005). Prolonged transcriptional silencing and CpG methylation induced by siRNAs targeted to the HIV-1 promoter region. J RNAi Gene Silencing.

[20] McBride K (2013). The majority of HIV type 1 DNA in circulating CD4 + T lymphocytes is present in non-gut-homing resting memory CD4 + T cells. AIDS Res Hum Retroviruses.

[21] Yamagishi M (2009). Retroviral delivery of promoter-targeted shRNA induces long-term silencing of HIV-1 transcription. Microbes Infect.

[22] Singh RK (2013). Kinetic and thermodynamic rationale for suberoylanilide hydroxamic acid being a preferential human histone deacetylase 8 inhibitor as compared to the structurally similar ligand, trichostatin A. Biochemistry.

[23] Thaler F, Mercurio C (2014). Towards selective inhibition of histone deacetylase isoforms: what has been achieved, where we are and what will be next. ChemMedChem.

[24] De Pablo-Bernal RS (2014). TNF-alpha levels in HIV-infected patients after long-term suppressive cART persist as high as in elderly, HIV-uninfected subjects. J Antimicrob Chemother.

[25] Diaz L (2015). Bryostatin activates HIV-1 latent expression in human astrocytes through a PKC and NF-kB-dependent mechanism. Sci Rep.

[26] Bernhard W (2011). The Suv39H1 methyltransferase inhibitor chaetocin causes induction of integrated HIV-1 without producing a T cell response. FEBS Lett.

[27] Friedman J (2011). Epigenetic silencing of HIV-1 by the histone H3 lysine 27 methyltransferase enhancer of Zeste 2. J Virol.

[28] Chen T, Dent SY (2014). Chromatin modifiers and remodellers: regulators of cellular differentiation. Nat Rev Genet.

[29] Damas P (1989). Tumor necrosis factor and interleukin-1 serum levels during severe sepsis in humans. Crit Care Med.

[30] Kim H (2010). Gene-specific RNA polymerase II phosphorylation and the CTD code. Nat Struct Mol Biol.

[31] Ng HH (2003). Targeted recruitment of Set1 histone methylase by elongating Pol II Provides a localized mark and memory of recent transcriptional activity. Mol Cell.

[32] Mehla R (2010). Bryostatin modulates latent HIV-1 infection via PKC and AMPK signaling but inhibits acute infection in a receptor independent manner. PLoS One.

[33] Ekinci FJ, Shea TB (1997). Selective activation by bryostatin-1 demonstrates unique roles for PKC epsilon in neurite extension and tau phosphorylation. Int J Dev Neurosci.

[34] Bertolotto C (2000). Protein kinase C theta and epsilon promote T-cell survival by a rsk-dependent phosphorylation and inactivation of BAD. J Biol Chem.

[35] Gutierrez-Uzquiza A (2015). PKCepsilon Is an essential mediator of prostate cancer bone metastasis. Mol Cancer Res.

[36] Spina CA (2013). An in-depth comparison of latent HIV-1 reactivation in multiple cell model systems and resting CD4 + T cells from aviremic patients. PLoS Pathog.

[37] Asiedu C (1995). Inhibition of leukemic cell growth by the protein kinase C activator bryostatin 1 correlates with the dephosphorylation of cyclin-dependent kinase 2. Cancer Res.

[38] Nekhai S (2002). HIV-1 Tat-associated RNA polymerase C-terminal domain kinase, CDK2, phosphorylates CDK7 and stimulates Tat-mediated transcription. Biochem J.

[39] Breuer D (2012). CDK2 regulates HIV-1 transcription by phosphorylation of CDK9 on serine 90. Retrovirology.

[40] Huang V (2013). Ago1 Interacts with RNA polymerase II and binds to the promoters of actively transcribed genes in human cancer cells. PLoS Genet.

[41] Allo M (2014). Argonaute-1 binds transcriptional enhancers and controls constitutive and alternative splicing in human cells. Proc Natl Acad Sci USA.

[42] Zhou F (2017). Identification of novel NF-kappaB transcriptional targets in TNFalpha-treated HeLa and HepG2 cells. Cell Biol Int.

[43] Zhou F (2014). NF-kappaB target microRNAs and their target genes in TNFalpha-stimulated HeLa cells. Biochim Biophys Acta.

[44] Xing Y (2013). Characterization of genome-wide binding of NF-kappaB in TNFalpha-stimulated HeLa cells. Gene.

[45] Guenther MG (2007). A chromatin landmark and transcription initiation at most promoters in human cells. Cell.

[46] Tee WW (2014). Erk1/2 activity promotes chromatin features and RNAPII phosphorylation at developmental promoters in mouse ESCs. Cell.

[47] Espinoza CA (2004). B2 RNA binds directly to RNA polymerase II to repress transcript synthesis. Nat Struct Mol Biol.

[48] Ponicsan SL (2013). The non-coding B2 RNA binds to the DNA cleft and active-site region of RNA polymerase II. J Mol Biol.

[49] Kobayashi-Ishihara M (2012). HIV-1-encoded antisense RNA suppresses viral replication for a prolonged period. Retrovirology.

[50] Saayman S (2014). An HIV-encoded antisense long noncoding RNA epigenetically regulates viral transcription. Mol Ther.

[51] Eekels JJ, Berkhout B (2011). Toward a durable treatment of HIV-1 infection using RNA interference. Prog Mol Biol Transl Sci.

[52] Wang G (2018). CRISPR-Cas based antiviral strategies against HIV-1. Virus Res.

[53] Xu B (2017). CRISPR/Cas9-mediated CCR5 ablation in human hematopoietic stem/progenitor cells confers HIV-1 resistance in vivo. Mol Ther.

[54] Kaminski R (2016). Excision of HIV-1 DNA by gene editing: a proof-of-concept in vivo study. Gene Ther.

[55] Limsirichai P, Gaj T, Schaffer DV (2016). CRISPR-mediated activation of latent HIV-1 expression. Mol Ther.

[56] Hu W (2014). RNA-directed gene editing specifically eradicates latent and prevents new HIV-1 infection. Proc Natl Acad Sci USA.

[57] Wang Z (2016). CRISPR/Cas9-derived mutations both inhibit HIV-1 replication and accelerate viral escape. Cell Rep.

[58] Kessing CF (2017). In vivo suppression of HIV rebound by didehydro-cortistatin A, a “block-and-lock” strategy for HIV-1 treatment. Cell Rep.

