# Supplementary material for: RNA-induced epigenetic silencing inhibits HIV-1 reactivation from latency
Source: Retrovirology. 2018 Oct 4;15:67. doi: 10.1186/s12977-018-0451-0 (PMC6172763; doi:10.1186/s12977-018-0451-0)
Supplement: Supplementary file 1 — Additional file 1. Statistical results of the ordinary two-way ANOVA analyses for all epigenetic marks. [file 12977_2018_451_MOESM1_ESM.docx]

**Additional file 1: Methods**

**Statistical Methods: Ordinary Two-way ANOVA**

| **TABLE A1.** Ordinary two-way ANOVA comparing epigenetic marks between cell lines and condition | | | | | | |
| --- | --- | --- | --- | --- | --- | --- |
| **Antibody against*^a^*** | **Interaction (F(Dfn, Dfd))*^b^*** | **P Value*^c^*** | **Cell line** | **P Value*^c^*** | **Condition (Latency vs Reactivation)** | **P Value*^c^*** |
| AGO1 | F (6, 42) = 34.60 | P < 0.0001 | F (6, 42) = 62.45 | P < 0.0001 | F (1, 42) = 376.0 | P < 0.0001 |
| HDAC1 | F (6, 42) = 19.95 | P < 0.0001 | F (6, 42) = 27.90 | P < 0.0001 | F (1, 42) = 69.58 | P < 0.0001 |
| H3K27me3 | F (6, 40) = 8.125 | P < 0.0001 | F (6, 40) = 6.998 | P < 0.0001 | F (1, 40) = 17.46 | P = 0.0002 |
| H3K9me2 | F (6, 42) = 3.467 | P =0.0071 | F (6, 42) = 10.88 | P < 0.0001 | F (1, 42) = 2.832 | P =0.095 |
| H3K9me3 | F (6, 39) = 2.593 | P = 0.0328 | F (6, 39) = 2.637 | P = 0.0304 | F (1, 39) = 24.72 | P < 0.0001 |
| H3K9Ac | F (6, 42) = 0.742 | P = 0.6189 | F (6, 42) = 2.606 | P = 0.0308 | F (1, 42) = 7.623 | P = 0.0085 |
| *^a^*Missing values: H3K27me3, 2; H3K9me3, 3. | | | | | | |
| *^b^*F: F distribution; Df: degrees of freedom; n: numerator; d: denominator. | | | | | | |
| *^c^*Threshold of significance for P values: p ≤ 0.05. | | | | | | |

| **TABLE A2.** Post hoc analyses: Holm - Šídák Multiple comparisons*^a^* | | | | | | | | | | | | |
| --- | --- | --- | --- | --- | --- | --- | --- | --- | --- | --- | --- | --- |
| **Condition*^b^*** | **H3K27me3** | | **H3K9me2** | | **H3K9me3** | | **H3K9Ac** | | **AGO1** | | **HDAC1** | |
|  | P value | Dir | P value | Dir | P value | Dir | P value | Dir | P value | Dir | P value | Dir |
| ***Latency*** | | | | | | | | | | | | |
| Parental vs. PromA | > 0.9999 | n/a | 0.9562 | n/a | > 0.9999 | n/a | 0.6248 | n/a | 0.0979 | n/a | 0.7405 | n/a |
| Parental vs. M2 | 0.9971 | n/a | 0.9172 | n/a | > 0.9999 | n/a | 0.0894 | n/a | 0.0126 | **dcr** | < 0.0001 | **dcr** |
| Parental vs. 143 | 0.3299 | n/a | 0.9974 | n/a | 0.9961 | n/a | 0.2996 | n/a | 0.9739 | n/a | 0.1294 | n/a |
| Parental vs. 143_3M | 0.9151 | n/a | 0.0349 | **dcr** | 0.6982 | n/a | 0.5306 | n/a | 0.005 | **dcr** | 0.0011 | **dcr** |
| Parental vs. A/143 | > 0.9999 | n/a | 0.9998 | n/a | 0.9989 | n/a | 0.2938 | n/a | 0.9883 | n/a | 0.059 | n/a |
| Parental vs. CtrL | 0.9998 | n/a | 0.9718 | n/a | 0.9982 | n/a | 0.1497 | n/a | 0.5054 | n/a | 0.0006 | **dcr** |
| ***Reactivation (TNF 5ng/mL)*** | | | | | | | | | | | | |
| Parental vs. PromA | 0.4692 | n/a | 0.472 | n/a | 0.8243 | n/a | 0.9998 | n/a | < 0.0001 | **inc** | 0.9998 | n/a |
| Parental vs. M2 | > 0.9999 | n/a | 0.3749 | n/a | 0.0207 | **dcr** | 0.1755 | n/a | 0.3946 | n/a | 0.8155 | n/a |
| Parental vs. 143 | 0.2709 | n/a | 0.0015 | **inc** | 0.1942 | n/a | 0.9697 | n/a | < 0.0001 | **inc** | < 0.0001 | **inc** |
| Parental vs. 143_3M | 0.9568 | n/a | 0.0141 | **dcr** | 0.6964 | n/a | 0.9599 | n/a | > 0.9999 | n/a | < 0.0001 | **dcr** |
| Parental vs. A/143 | < 0.0001 | **inc** | 0.8803 | n/a | 0.0143 | **dcr** | 0.605 | n/a | < 0.0001 | **inc** | < 0.0001 | **inc** |
| Parental vs. CtrL | 0.784 | n/a | 0.9773 | n/a | 0.0006 | **dcr** | 0.997 | n/a | 0.0021 | **inc** | 0.0935 | n/a |
| ***Latency vs Reactivation (TNF 5ng/mL)*** | | | | | | | | | | | | |
| PromA | 0.3566 | n/a | 0.9741 | n/a | 0.8708 | n/a | 0.8783 | n/a | < 0.0001 | **inc** | 0.9995 | n/a |
| M2 | > 0.999 | n/a | 0.9706 | n/a | 0.0125 | **dcr** | > 0.9999 | n/a | < 0.0001 | **inc** | 0.0112 | **inc** |
| 143 | 0.0037 | **inc** | 0.0004 | **inc** | 0.5143 | n/a | 0.8622 | n/a | < 0.0001 | **inc** | < 0.0001 | **inc** |
| 143_3M | > 0.9999 | n/a | > 0.9999 | n/a | > 0.9999 | n/a | 0.1322 | n/a | 0.0083 | **inc** | 0.0801 | n/a |
| A/143 | < 0.0001 | **inc** | 0.7486 | n/a | 0.0409 | **dcr** | 0.9992 | n/a | < 0.0001 | **inc** | < 0.0001 | **inc** |
| CtrL | 0.6329 | n/a | > 0.9999 | n/a | 0.0029 | **dcr** | 0.4392 | n/a | < 0.0001 | **inc** | < 0.0001 | **inc** |
| Parental | > 0.9999 | n/a | > 0.9999 | n/a | > 0.9999 | n/a | > 0.9999 | n/a | > 0.9999 | n/a | 0.8706 | n/a |
|  | | | | | | | | | | | | |
| *^a^*Analyses were performed on the data normalised to the Parental cell line using Holm Šídák Multicomparison test. Threshold of significance of adjusted P values: p ≤ 0.05. | | | | | | | | | | | | |
| *^b^*Dir: direction of the interaction; n/a: not applicable; inc: increase; dcr: decrease. | | | | | | | | | | | | |

| **TABLE A3.** Ordinary two-way ANOVA comparing between two epigenetic- or chromatin-associated marks “in-within” cell lines during Latency (GFP^-^)*^a^* | | | | | | | |
| --- | --- | --- | --- | --- | --- | --- | --- |
| **Antibody compared*^b^*** | **Interaction (F(Dfn, Dfd))*^c^*** | **P Value*^d^*** | **Cell line** | **P Value*^d^*** | **Condition** | **P Value*^d^*** |  |
| H3K4me3 vs H3K27me3 | F (6, 42) = 1.23 | P = 0.310 | F (6, 42) = 3.92 | P = 0.003 | F (1, 42) = 221.5 | P < 0.0001 |  |
| RNA PoL II pSer2 vs pSer5 | F (5, 36) = 2.98 | P = 0.024 | F (5, 36) = 2.74 | P = 0.034 | F (1, 36) = 95.13 | P < 0.0001 |  |
|  | | | | | | | |
| *^a^*Data analysed corresponded to the absolute values of the % of Input after subtracting the background signal from a No-antibody control. | | | | | | | |
| *^b^*pSer2:phosphorylated Serine 2; pSer5: phosphorylated Serine 5 | | | | | | | |
| *^c^*F: F distribution; Df: degrees of freedom; n: numerator; d: denominator. | | | | | | | |
| *^d^*Threshold of significance for P values: p ≤ 0.05. | | | | | | | |

| **TABLE A4.** HIV-1 Promoter bivalency and Phosphorylation of RNA Pol II | | | | |  |  |  |  |  |  |
| --- | --- | --- | --- | --- | --- | --- | --- | --- | --- | --- |
| **“*In-within*” Multiple comparisons*^a^*** | | | | |  |  |  |  |  |  |
| Latency*^b^* | H3K27me3 > H3K4me3 | | pSer2 > pSer5 | |  |  |  |  |  |  |
|  | P value | | P value | |  |  |  |  |  |  |
| PromA | < 0.0001 | | 0.0088 | |  |  |  |  |  |  |
| M2 | < 0.0001 | | 0.0486 | |  |  |  |  |  |  |
| 143 | < 0.0001 | | < 0.0001 | |  |  |  |  |  |  |
| 143_3M | < 0.0001 | | 0.0538 | |  |  |  |  |  |  |
| A/143 | < 0.0001 | | < 0.0001 | |  |  |  |  |  |  |
| CtrL | 0.0094 | | n/p | |  |  |  |  |  |  |
| Parental | < 0.0001 | | 0.0879 | |  |  |  |  |  |  |
| **“*In-between*” Multiple comparisons*^a^*** | | | | | | | | | | |
| Latency*^b^* | H3K4me3 | | | H3K27me3 | | | pSer2 | | pSer5 | |
|  | P value | Dir | | P value | | Dir | P value | Dir | P value | Dir |
| PromA vs. Parental | 0.9994 | n/a | | 0.9998 | | n/a | 0.9924 | n/a | 0.9972 | n/a |
| M2 vs. Parental | 0.9802 | n/a | | 0.9959 | | n/a | 0.9915 | n/a | 0.9523 | n/a |
| 143 vs. Parental | 0.3301 | n/a | | 0.3191 | | n/a | 0.028 | inc | 0.9862 | n/a |
| 143_3M vs. Parental | 0.5103 | n/a | | 0.0232 | | dcr | > 0.9999 | n/a | > 0.9999 | n/a |
| A/143 vs. Parental | 0.945 | n/a | | 0.607 | | n/a | 0.0086 | inc | 0.9983 | n/a |
| CtrL vs. Parental | 0.9954 | n/a | | 0.0616 | | n/a | np | np | np | np |
|  | | | | | | | | | | |
| *^a^*Pos Hoc Holm Šídák multiple comparison test was performed on absolute values of % of Input, normalised only by subtracting the background signal from a No-Antibody control. Dir: Direction of the interaction; dcr: decrease; inc: increase. Threshold of significance of adjusted P values: p ≤ 0.05. | | | | | | | | | | |
| *^b^*np: ChIP not performed; n/a : not applicable | | | | | | | | | | |
